# Inhibition of miR‐148a‐3p resists hepatocellular carcinoma progress of hepatitis C virus infection through suppressing *c‐Jun* and MAPK pathway

**DOI:** 10.1111/jcmm.14045

**Published:** 2018-12-18

**Authors:** Yibin Deng, Jianchu Wang, Meijin Huang, Guidan Xu, Wujun Wei, Houji Qin

**Affiliations:** ^1^ Clinic Medicine Research Center of Hepatobiliary Diseases The Affiliated Hospital of Youjiang Medical College for Nationalities Baise China; ^2^ Department of Infectious Diseases The Affiliated Hospital of Youjiang Medical College for Nationalities Baise China; ^3^ Centre for Medical Laboratory Science The Affiliated Hospital of Youjiang Medical College for Nationalities Baise China

**Keywords:** *c‐Jun*, hepatitis C virus, hepatoma, MAPK signalling pathway, miR‐148a‐3p

## Abstract

**Objectives:**

The present study was committed to investigate the role of miR‐148a‐3p in HCC infected with hepatitis C virus (HCV) and the regulatory mechanism of miR‐148a‐3p/*c‐Jun*/MAPK signalling pathway.

**Methods:**

Differential analysis and GSEA analysis were performed with R packages. QRT‐PCR and Western blot were used to detect RNA or protein level, respectively. The targeted relationship between miR‐148a‐3p and *c‐Jun* was predicted by TargetScan database and determined by double luciferase reporter assay. MTT assay and flow cytometry were used to evaluate cell proliferation, cell cycle and cell apoptosis, respectively.

**Results:**

*C* *‐Jun* was up‐regulated, and MAPK signalling pathway was activated in HCV‐infected HCC cells. *C‐Jun* expression regulated inflammation‐related gene expression and had an influence on cell proliferation, cell cycle and cell apoptosis. MiR‐148a‐3p, down‐regulated in HCV‐infected HCC cells, could target *c‐Jun* mRNA to suppress c‐Jun protein expression.

**Conclusions:**

MiR‐148a‐3p suppressed the proliferation of HCC cells infected with HCV through targeting *c‐Jun* mRNA.

## 1 INTRODUCTION

According to the data of International Agency for Research on Cancer in 2012, liver cancer was in the top 10 cancer sites that caused incident cases worldwide [https://gco.iarc.fr/]. Hepatitis C virus (HCV), identified as an enveloped positive RNA virus and a member of the family *Flaviviridae*, is a valid factor in a great diversity of human liver diseases, for instance, cirrhosis, liver fibrosis and hepatocellular carcinoma (HCC).^1^, ^2^, ^3^ According to the previous studies, HCV could be cleaved into four structural proteins (C, E1, E2 and p7) and six nonstructural proteins (NS2, NS3, NS4A, NS4B, NS4A and NS5B) by cellular and viral proteases.^1^, ^2^, ^4^ Among the 10 proteins, E2, which is considered as HCV envelope protein, has aroused the interests in the therapy of HCV. Zhao et  al revealed that E2 could stimulate the proliferation of human hepatoma cell via the MAPK/ERK signalling pathway in 2005.^5^ Results of Liu et  al displayed that synthesized peptides 705‐734 from E2 could induce the maturation through p38 MAPK signalling.^1^ The recent method of HCV therapy is direct‐acting antivirals (DAAs).^6^ However, uncertainty about the optimal timing of DAA therapy and other untoward response make more developed and efficient therapy of HCV urgent.

Mitogen‐activated protein kinase (MAPK) family, a group of serine/threonine kinases, plays an essential role in the regulation of the cell activities, for instance, apoptosis, cell cycle and transcription. It could be divided into three group—p38 family kinases, c‐Jun N‐terminal kinase (*JNK*) and extracellular signal‐regulated kinase (ERK).^3^, ^5^, ^7^ Many researches have been implemented to uncover the specific mechanism of MAPK. Besides the studies mentioned above in HCV, MAPK signalling pathway could also be carried out in other fields.^8^, ^9^ For example, data in the study of Li et  al showed that Angiotensin II regulated the expression of miR‐143/145 through p38 MAPK signalling pathway in the pathogenesis of aortic dissection.^9^ However, the mechanisms of MAPK signalling pathway remain to be explored and supplemented.

*C‐Jun*, a member of the activating protein 1 (AP‐1) transcription factor family, has been proved to have a significant role in cellular processes, such as apoptosis, migration and differentiation.^10^ Mariani demonstrated that Jun oncogene was high expressed in high aggressive sarcomas and could block adipocytic differentiation.^11^ Zenz revealed that *c‐Jun* had a regulation on the development of eyelid closure and skin tumour via EGFR signalling.^12^ Results of Liu et  al displayed the function of HP1a/KDM4A in the mechanism of *c‐Jun* regulation.^10^

MiRNA is a length of 19‐25 nt (nucleotides) noncoding RNAs.^13^ MiRNAs have a regulation in cell proliferation, differentiation, apoptosis and other cellular processes via enhancing or inhibiting the expression of many mRNAs.^14^, ^15^, ^16^ Numerous previous studies have manifested that miR‐148a‐3p was involved in laryngeal squamous cell carcinoma (LSCC), bladder cancer, gastric cancer and other several cancers.^13^, ^14^, ^15^ However, there is a gap in the study of miR‐148a‐3p in the field of HCV, especially related to miRNA.

In the current study, we found that miR‐148a‐3p was expressed remarkably low and MAPK signalling pathway was activated through exploring the influences of HCV on the infection cells. Further, we tried to confirm the regulation of miR‐148a‐3p on HCV infection and the internal mechanism.

## 2 MATERIALS AND METHODS

### 2.1 Patients

Healthy individuals (normal, n = 15), hepatitis patients infected with hepatitis C virus (HCV, n = 15) and HCC patients infected with HCV (HCC, n = 15) were recruited from the Affiliated Hospital of Youjiang Medical College for Nationalities in this study. The patients’ clinical characteristics are provided in Table S1. All patients and healthy individuals obtained their written informed consent, and this study protocol was approved by the Affiliated Hospital of Youjiang Medical College for Nationalities. Blood was collected from the cubital vein with anticoagulant (heparin sodium) and processed immediately.

### 2.2 Cell culture and infective assay

Human HCC cell lines HLE, HepG2, Huh‐7, Huh‐7.5.1 and SK‐HEP‐1 were purchased from the BeNa Culture Collection (Peking, China). HLE and SK‐HEP‐1 cells were incubated in Roswell Park Memorial Institute (RPMI‐1640) with 10% foetal bovine serum (FBS) (HyClone, South Logan, UT, USA). HepG2, Huh‐7 and Huh‐7.5.1 cells were cultured in high‐glucose Dulbecco's modified Eagle's medium (DMEM‐H) with 10% FBS. All cells were cultured at 37°C in a humidified incubator with 5% CO_2_. The Huh‐7.5.1 cells were infected with HCV (JFH‐1 strain; MOI = 0.1‐10) and propagated for 10 days. Stock virus was prepared by collecting and filtering the cell culture supernatant and was stored at −80°C until use. Viral RNA in the cell culture medium was isolated with the RNA pure Virus Kit (CW Biotech, Beijing, China), and HCV RNA replicates were quantified by qRT‐PCR described as follows.

### 2.3 Microarray analysis

The expression profile GSE44210 (GPL6480) was derived from gene expression omnibus (GEO) database to analyse differentially expressed genes (DEGs) in HCV‐infected Huh‐7.5.1 cells. INF11‐INF14 (infected groups) total samples and NI11‐NI14 (noninfected group) total samples were selected to screen differentially expressed mRNAs and miRNAs with Limma package (fold changes >2.0 and *P* adjust < 0.05). The miRNAs that targeted *c‐Jun* were predicted by TargetScan database (https://www.targetscan.org/vert_71/).

### 2.4 Gene set enrichment analysis (GSEA)

The enrichment analysis for KEGG pathway was performed with the normalized mRNA expression profiles of GSE44210 by GSEA v3.0 software. The enriched pathways were visualized by dotplot and gesaplot with ggplot2, grid, devtools and easygplot2 packages.

### 2.5 Cell transfection

MiR‐148a‐3p mimics and miR‐148a‐3p inhibitors (final concentration, 50 nmol/L; Ribobio, Guangzhou, China) were transfected into Huh‐7.5.1 cells for miR‐148a‐3p high expression or low expression, respectively. All miRNAs were purchased from Ribobio. SiRNA 1 and siRNA 2 for silencing *c‐Jun* (si‐*c‐Jun*‐1 and si‐*c‐Jun*‐2) were purchased from Ribobio. The sequences of miRNA mimics, inhibitor and siRNA are in Table 1. Transfection was performed by Lipofecta‐mine 2000 (Invitrogen, Shanghai, China) after Huh‐7.5.1 cells infected HCV 3 days. All siRNAs were tested and verified to reduce expression in Huh‐7.5.1 cells by Western blot analysis (>50% protein reduction) or qRT‐PCR (>80% mRNA).

**Table 1:** miRNA mimics and inhibitor, *c‐Jun* siRNA sequence

Subject	Sequence (5′→3′)
miR‐148a‐3p mimics	UCAGUGCACUACAGAACUUUGU
miR‐148a‐3p inhibitor	AGUCACGUGAUGUCUUGAAACA
Si‐c‐Jun 1	GGAUCAAGGCGGAGAGGAA
Si‐c‐Jun 2	GCAAACCUCAGCAACUUCA

### 2.6 qRT‐PCR

Total RNA was extracted from cells with TRIzol reagent (Invitrogen) in the light of the manufacturer's instructions. Quantitative RT‐PCR (qPCR) analysis was performed using the Roche Light Cycler 480 and SYBR RT‐PCR kits (DBI Bioscience, Shanghai, China); each 20 mL reaction contained 0.5 mmol/L of each PCR primer (Table 2), 10 mL of SYBR Green PCR master mix, 1 mL of diluted template and RNase‐free water. Target gene expression was standardized by glyceraldehyde 3‐phosphate dehydrogenase (GAPDH) expression. Quantification of miRNAs was performed by qRT‐PCR using miRNA analysis kits (Applied Biosystems, Shanghai, China) in the light of the manufacturer's instructions. The relative expression of miRNAs was normalized to that of internal control U6 snRNA within each sample using the 2^‑ΔΔCt^ method.

**Table 2:** PCR primer sequence

Name	Forward primer (5′→3′)	Reverse primer (5′→3′)
miR‐148a‐3p	TCAGTGCACTACAGAACTTTGT	GAATACCTCGGACCCTGC
c‐Jun	TCCTGCCCAGTGTTGTTTGT	GACTTCTCAGTGGGCTGTCC
IL‐6	CTTCTCCACAAACATGTAACAAGAG	TGGCATTTGTGGTTGGGTCA
MMP‐9	TCTATGGTCCTCGCCCTGAA	TTGTATCCGGCAAACTGGCT
MMP‐13	CCCCAGGCATCACCATTCAA	CATCAGGAACCCCGCATCTT
TNF‐α	ACTTCCTTGAGACACGGAGC	CTTCCCACCCACAAGAAGAGG
IL‐1α	CGTTTTGACGACGCACTTGT	GCCGTGAGTTTCCCAGAAGA
U6	CTCGCTTCGGCAGCACATA	AACGATTCACGAATTTGCGT
GAPDH	TTCTGGGATACACGGAGCAC	TACCAGCACCAGCGTCAAAG

### 2.7 Western blot

For Western blot assay, whole‐cell lysates were prepared by lysing cells with RIPA (PBS [pH 7.4] containing 0.01% Triton‐100, 0.01% EDTA and 10% cocktail protease inhibitor [Roche, Basel, Switzerland]). Equal amounts of proteins were determined by the Bradford assay (Bio‐Rad, Redmond, WA, USA). Cell lysates (100 mg) were electrophoresed by 12% SDS‐PAGE and then transferred to the PVDF membrane (Millipore, Marlborough, MA, USA). The membranes were blocked with 5% nonfat dried milk before incubating with specific primary antibodies. Membranes were then washed four times with PBS Tween‐20 and newly incubated with peroxidase‐coupled secondary antibodies. After incubation, protein bands were visualized with enhanced chemiluminescence (ECL) (Amersham Pharmacia Biotechnology, Freiburg, Germany).

### 2.8 MTT assay

Cell viability was quantified by MTT assay. After transfecting with *c‐Jun* siRNAs or miR‐148a‐3p mimics or inhibitor 24 hours, cells were resuspended and then 2000 cells were seeded in 96‐well plates. At 0 hour, 12 hours, 24 hours, 36 hours and 48 hours, cells were treated with MTT reagent (100 μL of fresh serum‑free medium with 0.5 g/L of MTT) at 37°C for 4 hours, following by adding with 50 μL DMSO each well for 10 minutes. The absorbance of each well was measured by microplate photometer at 450 nm.

### 2.9 Flow cytometry analysis

For cell cycle, propidium iodide (PI) staining was used. Cells were seeded in six‐well culture plates and cultured to 80% confluence before transfected with si‐*c‐Jun* RNAs or miR‐148a‐3p mimics and inhibitor for 24 hours. Cells were treated with PI and RNase for 30 minutes in the dark. Cell cycle was analysed by flow cytometry (FACSCalibur; Becton Dickinson, Franklin Lakes, NJ, USA).

For cell apoptosis, Annexin V‐Fluorescein Isothiocyanate (FITC)/PI apoptosis detection kit (BD Biosciences, Franklin Lakes, NJ, USA) was performed in accordance with the manufacturer's instruction. Cells were washed with PBS and resuspended with staining buffer. 5 μL Annexin V and 5 μL PI were added into a total of 100 μL cell suspension. The mix was incubated for 20 minutes at room temperature in the dark and finally subjected to analyse the proportion of apoptotic cells by flow cytometry.

### 2.10 Double luciferase reporter assay

Through the TargetScan database, it was predicted that the *c‐Jun* mRNA 3′UTR had seed sequences which were partially complementary to miR‐148a‐3p. *C‐Jun* 3′UTR fragments and their mutants were cloned the downstream of the luciferase coding region in the pGL3‐basic (Promega, Beijing, China) to construct pGL3‐*c‐Jun*‐3’UTR wild‐type and mutant type. Huh‐7.5.1 cells were cotransfected with pGL3‐*c‐Jun*‐3’UTR wild‐type (pGL3‐*c‐Jun*‐3’UTR mutant type) with miR‐148a‐3p mimics or its negative control. After 24 hours, luciferase activities were determined with the Dual‐Luciferase Reporter Assay System (Promega) in the light of the manufacturer's protocol.

### 2.11 Ad‐HCV core adenovirus construction and infection

The Ad easy system (Invitrogen; Thermo Fisher Scientific, Inc, Waltham, MA, USA) was used to construct the Ad HCV core adenovirus and the control Ad enhanced green fluorescent protein (EGFP) adenovirus. Huh‐7.5.1 cells infected with the Ad‐HCV core adenovirus and the control Ad‐EGFP adenovirus were incubated in 6‐well plate for 48 hours and then were collected to analyse the total RNA and protein expression levels.

### 2.12 Enzyme‐linked immunosorbent assay (ELISA)

Huh‐7.5.1 cells infected with JFH1 were seeded into 24‐well plates. Culture supernatant fluids were collected at the indicated time periods. And then HCV core antigen level in the cell culture supernatant fluids were quantified with the HCV core antigen ELISA kits (Ortho‐Clinical Diagnostics, Raritan, NJ, USA) in the light of the manufacturer's instructions.

### 2.13 Statistical analysis

GraphPad Prism 6.0 software (GraphPad Software, San Diego, CA, USA) is used to analyse the results, and the results are showed with mean ± SD. All the experiments were repeated three times. Statistical analysis of differences is performed by one‑way analysis of variance (ANOVA) or Student's *t* test. A statistically significant difference appeared with *P < 0.05.

## 3 RESULTS

### 3.1 HCV up‐regulated *c‐Jun* and activated MAPK pathway in Huh7 cells

**Figure 1:**
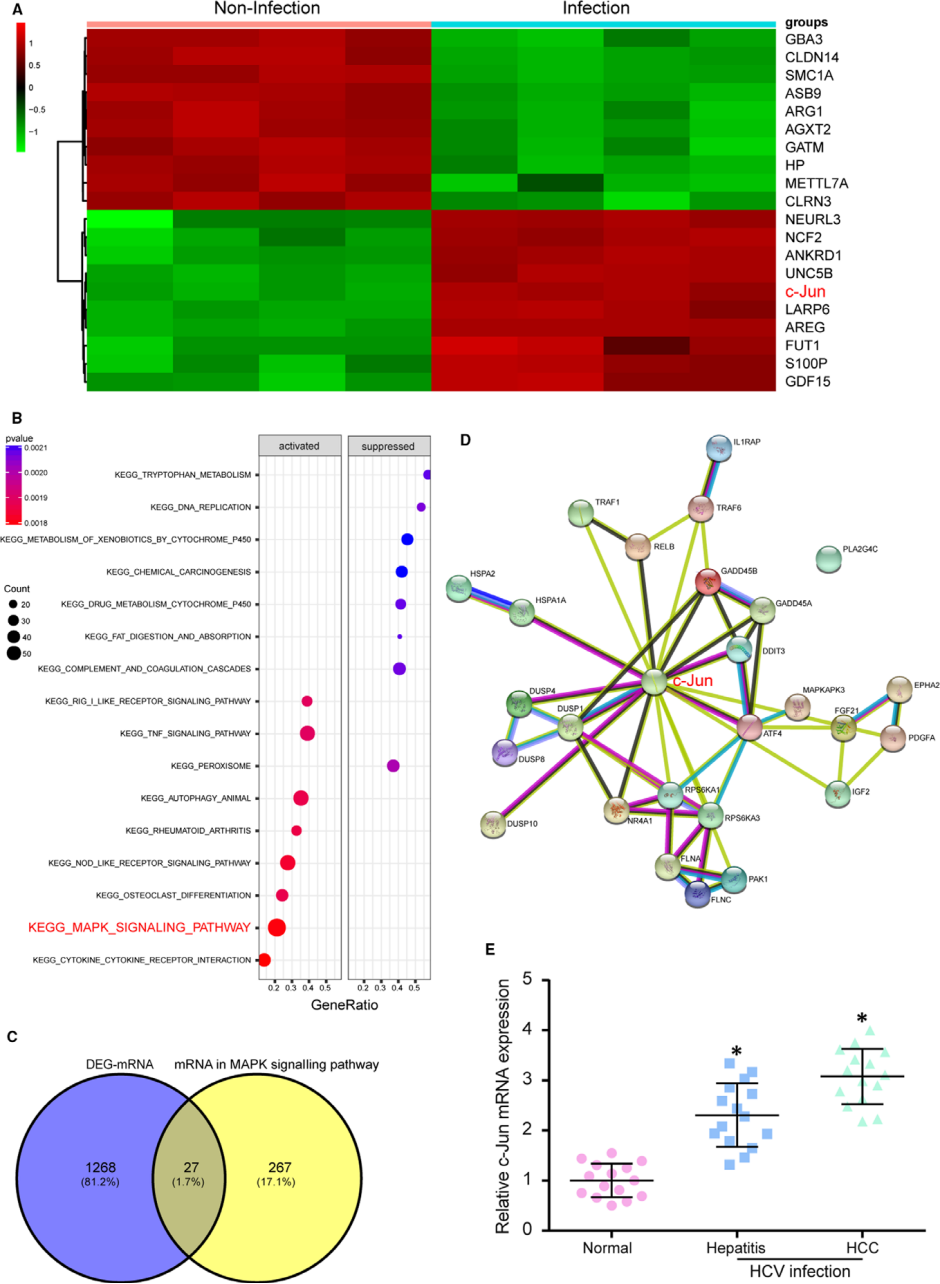
*C‐Jun* was overexpressed in infection with HCV host and MAPK signalling pathway was activated. A, The expression of *c‐Jun* was significantly higher in HCV infection host than noninfection host showed by heat map. B, The dotplot showed MAPK signalling pathway was activated in HCV infection host. C, There are 27 intersecting genes between mRNA difference analysis and mRNA in MAPK signalling pathway. D, The gene‐*c‐Jun* was in the centre of 27 intersecting genes by the STRING website. E, The result of qRT‐PCR showed that *c‐Jun* was highly expressed in hepatitis and HCC infected with HCV compared with that in normal people. *P < 0.05, compared with normal people

Limma packages were applied to significance analysis of differentially expressed genes (DEGs) between JFH1‐infected and uninfected Huh‐7.5.1 cell from GSE44210. The criteria were set as follows for screening differential genes in two groups: *P* < 0.05 and the absolute value of logFC >2. The cluster analysis was performed, and the heat map was drawn according to their expression levels. As shown in Figure 1A, C*‐Jun* was up‐regulated in HCV infection group. Using GSEA for KEGG pathway analyses, we identified several vital pathways involved in HCC infected with HCV. The dotplot picture showed that differential mRNAs were significantly enriched in eight pathways (*P* < 0.05), including the activated pathway‐MAPK signalling pathway (Figure 1B). Then, the Venn diagram showed that there were 27 common mRNAs between differential expression mRNAs in JFH1‐infected Huh‐7.5.1 cell and MAPK signalling pathway related mRNAs (Figure 1C). These common genes are showed in Table S2. STRING online retrieval site was used to uncover the association between these genes, and *c‐Jun* turned out to be in the centre of 27 intersecting genes, which indicated that it played vital and stable roles in HCV infection (Figure 1D). The *c‐Jun* mRNA expression level was higher in hepatitis and HCC infected with HCV compared with those in normal people (Figure 1E, *P* < 0.05).

### 3.2 Determination of the *c‐Jun* expression during HCV infection in HCC cells

**Figure 2:**
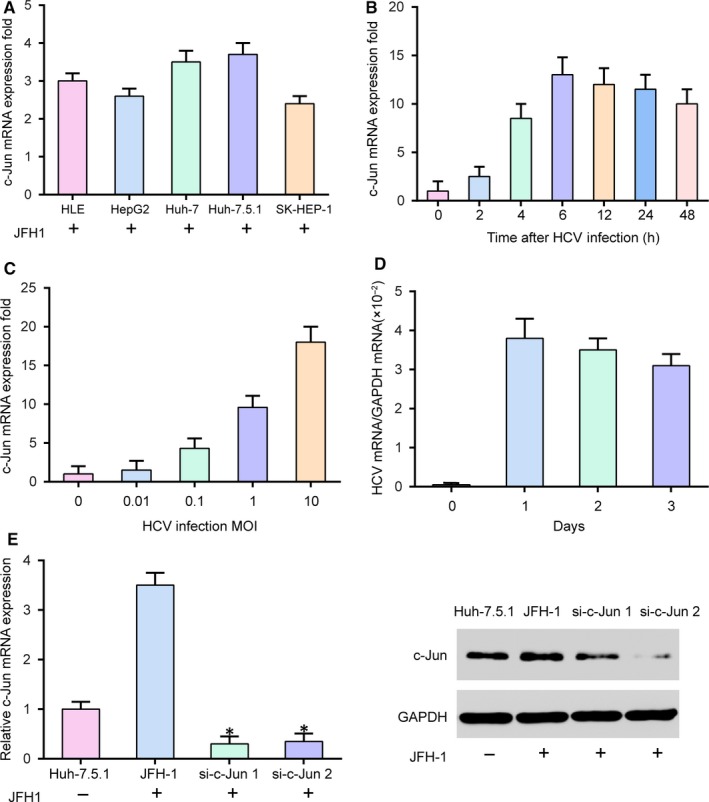
Determination of the expression of *c‐Jun* during HCV infection in HCC cells. A, *C‐Jun* mRNA was detected by qRT‐PCR in different hepatoma cells after infecting HCV. B, Human HCC cell line Huh‐7.5.1 was infected with or without HCV (MOI = 1) for different times as indicated. The expression of *c‐Jun* mRNA was measured by qRT‐PCR. Results are standardized to 1 in uninfected cells. C, Huh‐7.5.1 cells were infected with or without HCV at different MOIs, as indicated, for 12 h, and expression of *c‐Jun* mRNA was determined by qRT‐PCR. D, The amount of HCV mRNA in Huh‐7.5.1 cell was measured by qRT‐PCR. HCV RNA was expressed in cells at least three days after transfection with the HCV RNA. E, *C‐Jun* expression level was down‐regulated by si‐*c‐Jun*. JFH‐1, Huh‐7.5.1 cell infected with the JFH‐1 strain of HCV. *P < 0.05, compared with JFH‐1

As shown in Figure 2A, the results suggested that *c‐Jun* expression level in Huh‐7.5.1 cell line was the highest among the HCC cell lines (Huh‐7, HLE, HepG2 and SK‐HMP‐1) infected with JFH1. Therefore, subsequent studies were conducted using Huh‐7.5.1 cell line. The relationship between HCV infection and *c‐Jun* expression was determined through detecting *c‐Jun* mRNA levels in Huh‐7.5.1 cell infected with HCV strain JFH1. The results in uninfected cells were standardized to 1. The expression levels of *c‐Jun* were notably increased in Huh‐7.5.1 cell after infection HCV and reached a peak at 6 hours (Figure 2B, *P* < 0.05). Human Huh‐7.5.1 cell line was infected without or with HCV at different MOIs, and the expression of *c‐Jun* was detected by qRT‐PCR. We discovered that *c‐Jun* expression levels in Huh‐7.5.1 cells were increased with the increase of infection HCV MOIs (Figure 2C). The amount of HCV mRNA showed that the HCV RNA expressed stably at least 3 days after transfection with HCV RNA (Figure 2D). *C‐Jun* expression level was down‐regulated by si‐*c‐Jun* (Figure 2E, *P* < 0.05).

### 3.3 *C‐Jun* promoted related protein expression and cell proliferation, regulated cell cycle and inhibited apoptosis

**Figure 3:**
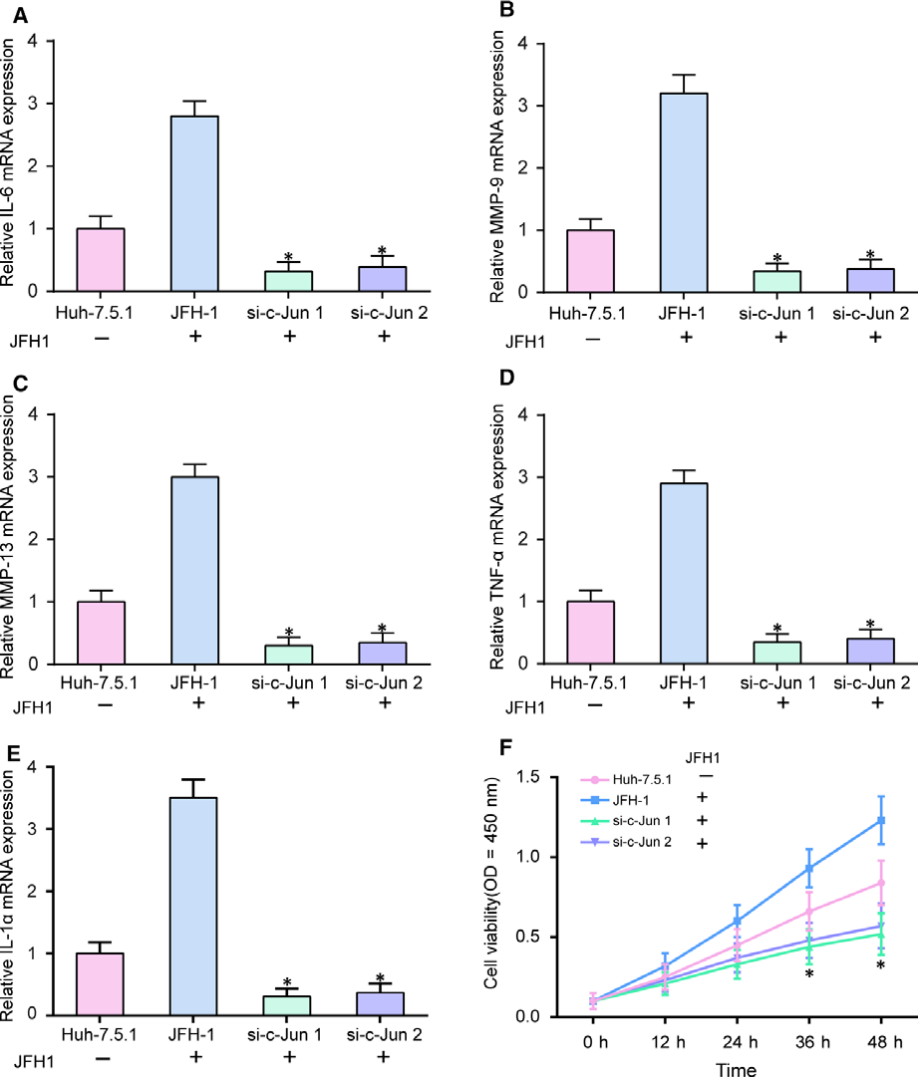
*C‐Jun* promoted related protein expression and cell proliferation. A‐E, Expressions of *IL‐6*, *MMP‐9*, *MMP‐13*, *TNF‐α* and *IL‐1α* mRNA were detected by qRT‐PCR. F, MTT assay results showed that si‐*c‐Jun* suppressed cell proliferation. JFH‐1, Huh‐7.5.1 cell infected with the JFH‐1 strain of HCV. *P < 0.05, compared with JFH‐1

**Figure 4:**
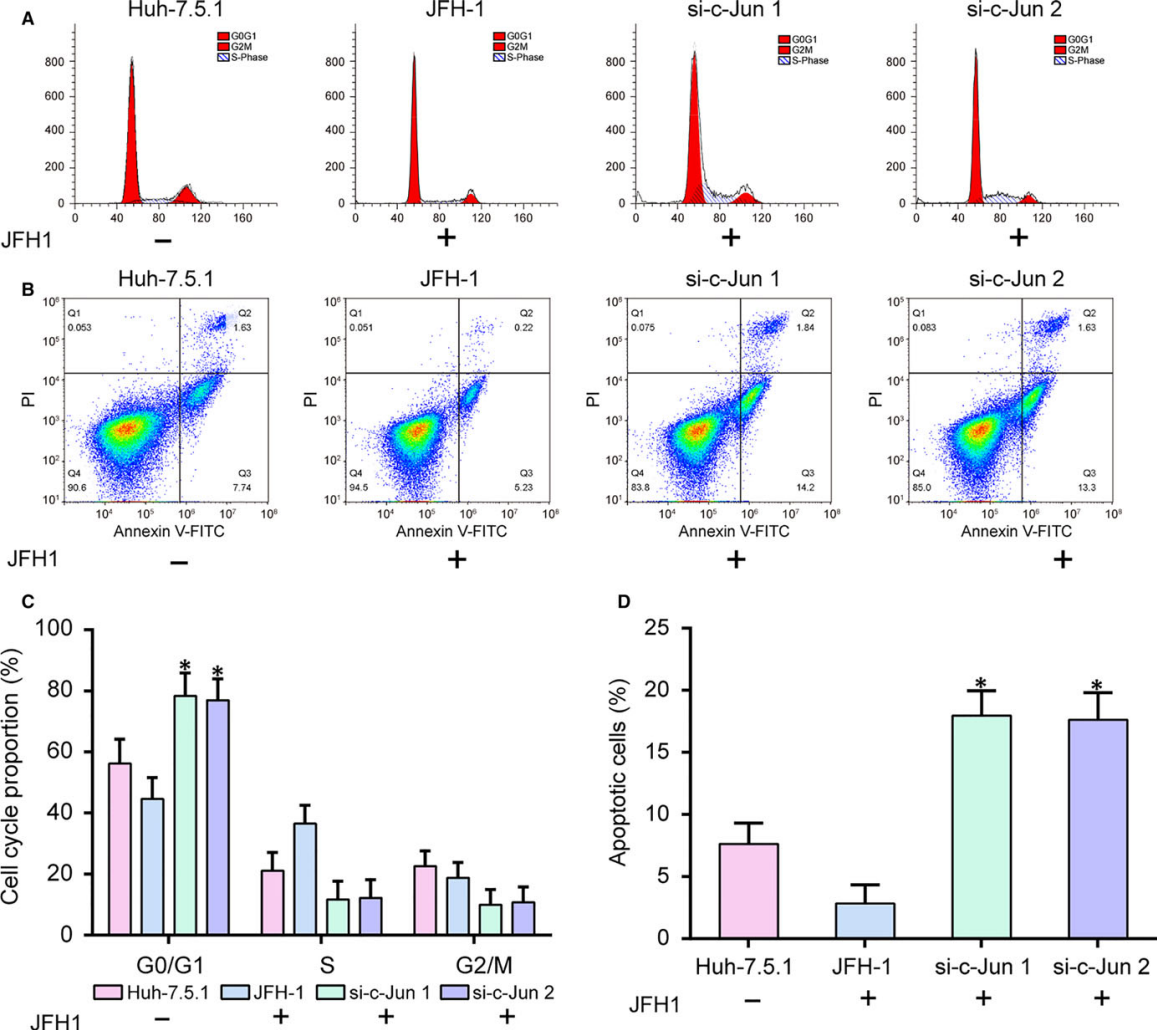
*C‐Jun* regulated cell cycle and inhibited apoptosis. A and C, The cell cycle was blocked in the G1 phase by down‐regulating *c‐Jun*. B and D, Cell apoptosis was promoted through inhibiting *c‐Jun* expression. JFH‐1, Huh‐7.5.1 cell infected with the JFH‐1 strain of HCV. *P < 0.05, compared with JFH‐1

Expression levels of inflammation‐related mRNAs *IL‐6*, *MMP‐9*, *MMP‐13*, *TNF‐α* and *IL‐1α* were detected by qRT‐PCR. As shown in Figure 3A‐E (*P* < 0.05), the expression levels were all decreased in si‐*c‐Jun* groups, suggesting that the low expression level of *c‐Jun* could alleviate inflammatory response. The effect of *c‐Jun *on the viability of Huh7.5.1 cells was analyzed by MTT assay (Figure 3F, *P* < 0.05)***.*** The viability of Huh‐7.5.1 cells infection with JFH1 in si‐*c‐Jun* groups was decreased, suggesting that *c‐Jun* could promote cell viability. Then, flow cytometry was performed to detect the changes of cell cycle and apoptotic rate in 4 groups (Figure 4A‐D). The results revealed that cell cycle was blocked in the G1 phase by down‐regulating *c‐Jun* (Figure 4A,C, *P* < 0.05). The apoptosis rates of cells were higher in si‐*c‐Jun* groups compared with that in JFH‐1 group (Figure 4B,D, *P* < 0.05). All of the results indicated that *c‐Jun* stimulated proliferation and suppressed cell cycle and apoptosis.

### 3.4 There was a targeted relationship between miR‐148a‐3p and *c‐Jun*

**Figure 5:**
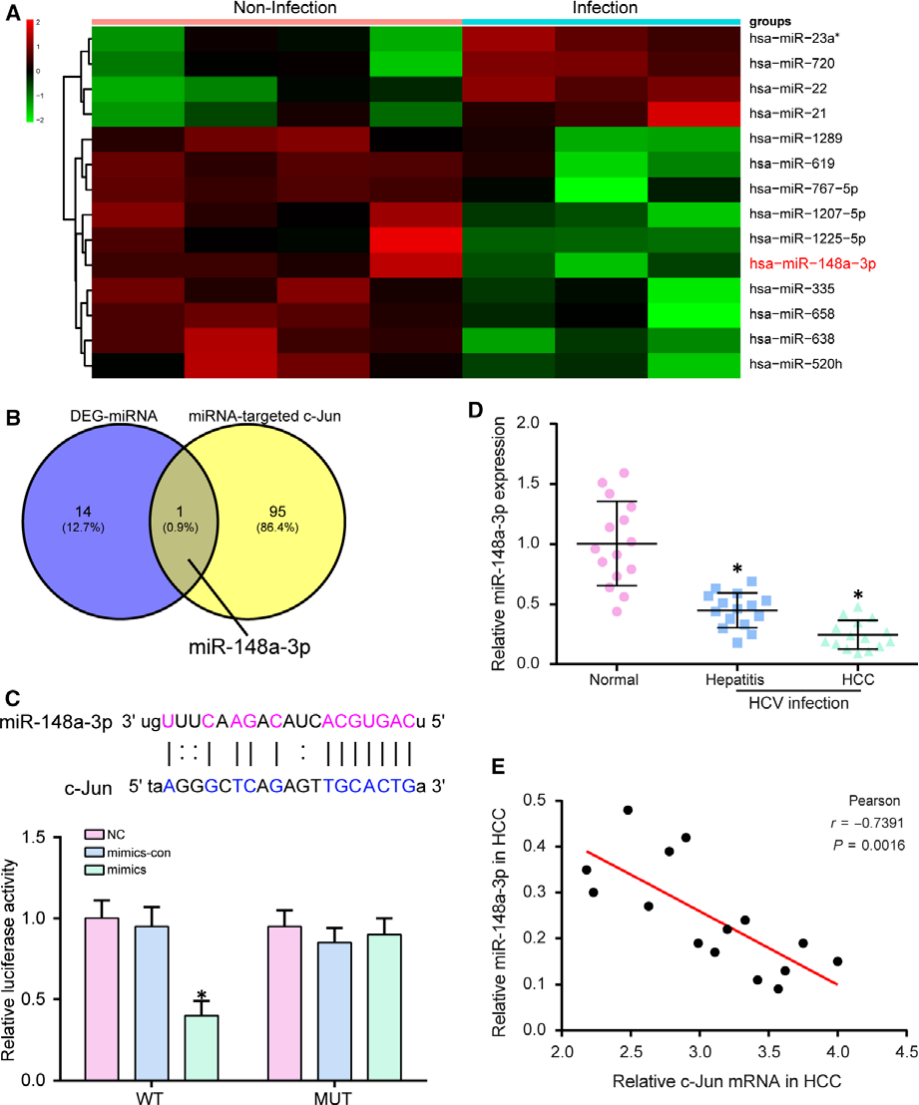
There was a targeted relationship between miR‐148a‐3p and *c‐Jun*. A, The heat map showed miR‐148a‐3p was down‐regulated in HCV infection host. B, MiR‐148a‐3p was the only one intersecting miRNA of differential expression miRNAs in HCV‐infected host and miRNAs targeted *c‐Jun*. C, There was the potential target between miR‐148a‐3p and *c‐Jun*. The targeted relationship between miR‐148a‐3p and *c‐Jun* was verified by Luciferase Reporter Assay. D, There was a lower expression level of miR‐148a‐3p in hepatitis and HCC infected with HCV compared with that in normal people. E, There was a negative correlation between miR‐148a‐3p and *c‐Jun*. Pearson correlation coefficient *r* = 0.7391. *P < 0.05, compared with normal people

Expression levels of miRNAs were considered to be significantly different if their levels meet the standard |log_2_(FC)|>1 and *P*. adjust < 0.05. Figure 5A showed the top four miRNAs which were up‐regulated in infection HCV group and the top 10 down‐regulated miRNAs including miR‐148a‐3p. According to the Venn diagram in Figure 5B, there was only one of the miRNAs targeted by *c‐Jun* that was differentially expressed in the host of HCV infection. The potential targeted relationship between miR‐148a‐3p and *c‐Jun* was predicted by TargetScan and verified by Luciferase Reporter Assay (Figure 5C). The results revealed that the luciferase activity of cells cotransfected with miR‐148a‐3p mimics and *c‐Jun* 3’UTR‐WT was significantly lower than that of cells cotransfected with miR‐148a‐3p mimics and *c‐Jun* 3’UTR‐MUT. Furthermore, qRT‐PCR result revealed that miR‐148a‐3p level was lower in hepatitis and HCC infection with HCV than that in normal people (Figure 5D, *P* < 0.05). The Pearson correlation coefficient showed there was a negative correlation between miR‐148a‐3p and *c‐Jun* expression in HCC infected with HCV (Figure 5E, *P* = 0.0016). We constructed the Ad‐HCV core adenovirus for infection Huh‐7.5.1 cells to overexpress HCV core protein and subsequently detected the expression of HCV core protein, *c‐Jun *and miR‐148a‐3p. As shown in Figure S1A‐S1D, HCV core protein and *c‐Jun* were up‐regulated in Ad‐HCV core infected cells, whereas miR‐148a‐3p was down‐regulated.

### 3.5 MiR‐148a‐3p regulated the expression of *c‐Jun*, IL‐6, MMP‐9, MMP‐13, TNF‐α and IL‐1α mRNA and affected proliferation, cell cycle and apoptosis

**Figure 6:**
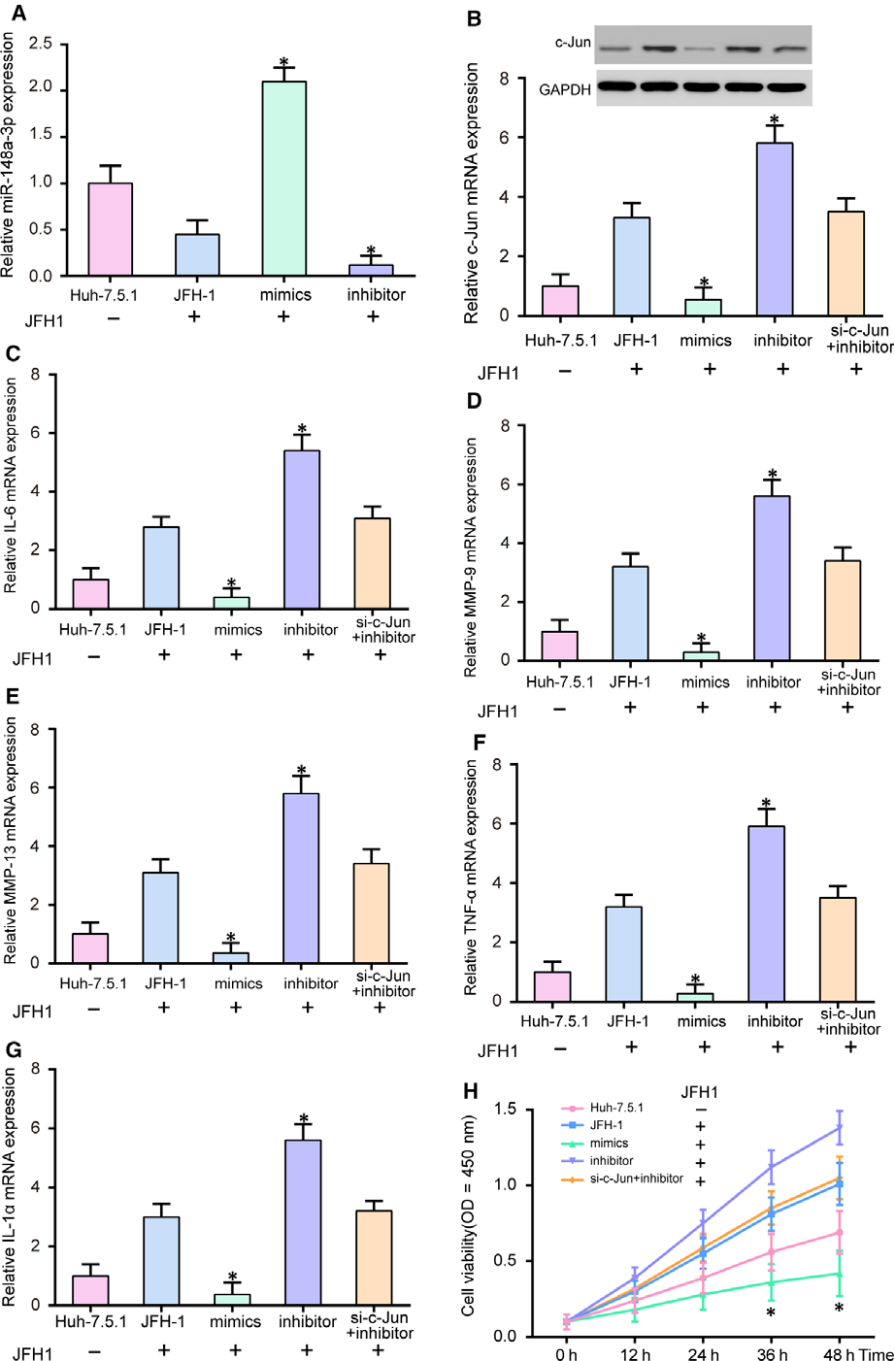
MiR‐148a‐3p regulated the expression of *IL‐6*, *MMP‐9*, *MMP‐13*, *TNF‐α* and *IL‐1α* mRNA and affected proliferation. A, MiR‐148a‐3p expression was up‐regulated by miR‐148a‐3p mimics and down‐regulated by miR‐148a‐3p inhibitor. B, MiR‐148a‐3p mimics could suppress *c‐Jun* expression while miR‐148a‐3p inhibitor had the opposite effect. C‐G, Expressions of *IL‐6*, *MMP‐9*, *MMP‐13*, *TNF‐α* and *IL‐1α* mRNA were detected by qRT‐PCR. H, MiR‐148a‐3p inhibitor promoted proliferation, and miR‐148a‐3p mimics suppressed proliferation while cotransfection si‐*c‐Jun* and inhibitor could restore the effect of miR‐148a‐3p. JFH‐1, Huh‐7.5.1 cell infected with the JFH‐1 strain of HCV. *P < 0.05, compared with JFH‐1

**Figure 7:**
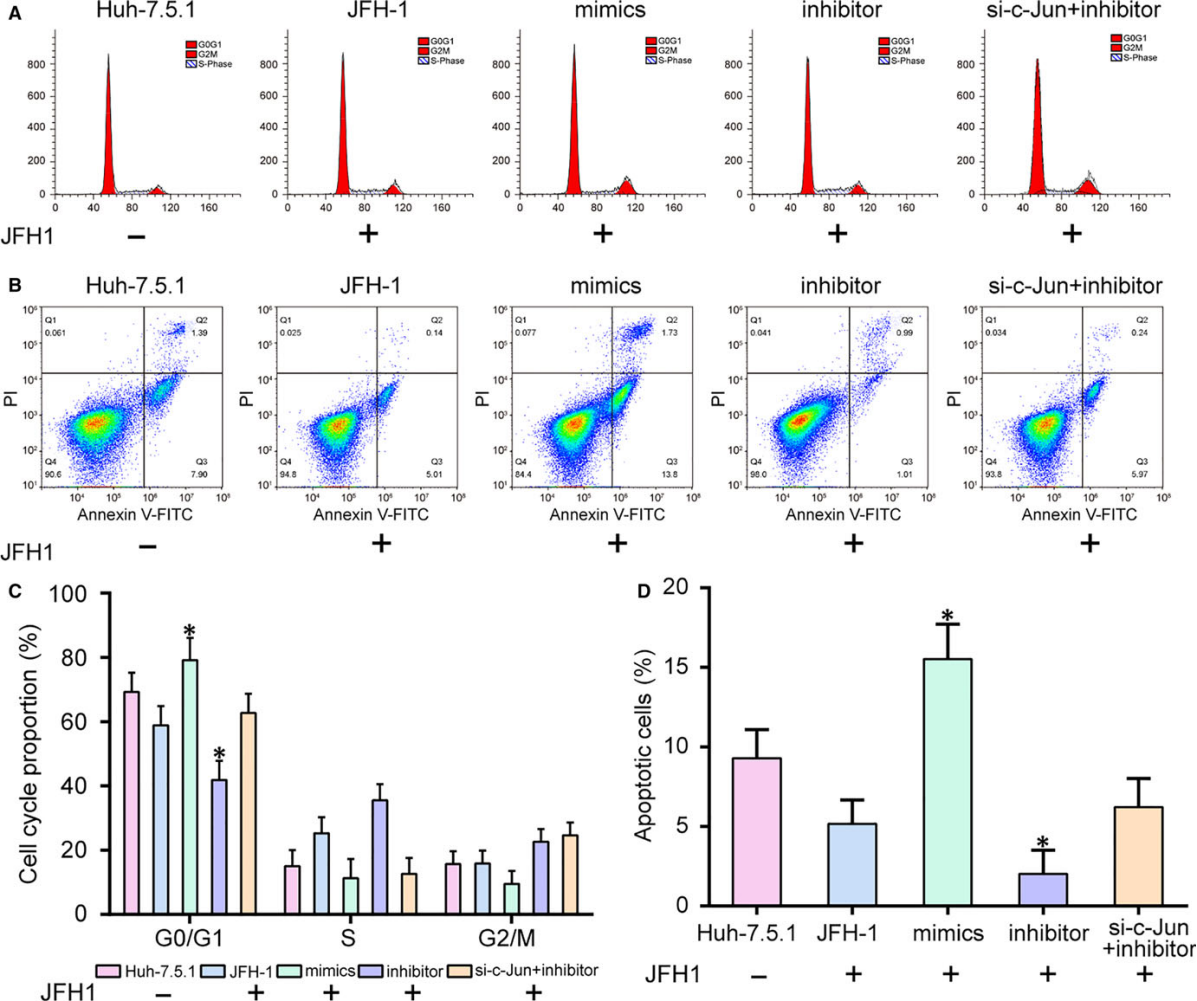
MiR‐148a‐3p affected cell cycle and apoptosis. A and C, MiR‐148a‐3p inhibitor blocked the cell cycle in G1 phase. B and D, Cell apoptosis was promoted by miR‐148a‐3p mimics. JFH‐1, Huh‐7.5.1 cell infected with the JFH‐1 strain of HCV. *P < 0.05, compared with JFH‐1

The transfection efficiency of miR‐148a‐3p was shown in Figure 6A (*P* < 0.05). MiR‐148a‐3p expression level was notably elevated in mimics group while decreased in inhibitor group when compared to JFH‐1 group. *C‐Jun* mRNA and protein expression levels were inhibited by miR‐148a‐3p mimics but was facilitated by miR‐148a‐3p inhibitor (Figure 6B, *P* < 0.05). As shown in Figure 6C‐G, the expression levels of inflammation‐related *IL‐6*, *MMP‐9*, *MMP‐13*, *TNF‐α* and *IL‐1α* mRNA were all increased by miR‐148a‐3p inhibitor (*P* < 0.05). No significantly difference was found between si‐*c‐Jun*+inhibitor group and JFH‐1 group. These results suggested that the inhibitor of miR‐148a‐3p could aggravate inflammatory response, while the stimulatory effect could be neutralized by simultaneously knockdown of *c‐Jun*. Compared with control group, the viability of cells in inhibitor group was enhanced, while miR‐148a‐3p mimics suppressed cell viability (Figure 6H, *P* < 0.05). Then, flow cytometry was used to detect cell cycle and apoptotic rate in four groups (Figure 7A‐D). The results revealed that cell cycle was blocked in the G1 phase by miR‐148a‐3p (Figure 7A,C, *P* < 0.05). The apoptosis rates were higher in miR‐148a‐3p mimics groups while lower in miR‐148a‐3p inhibitor group (Figure 7B,D, *P* < 0.05). All the results indicated that miR‐148a‐3p could affect the biological function of HCC cells infected with HCV by inhibiting *c‐Jun* expression. For example, miR‐148a‐3p could alleviate inflammatory reaction, suppress proliferation and cell cycle, and induce apoptosis.

## 4 DISCUSSION

In this study, we suggested that hepatitis C virus (HCV) could enhance the expression of *c‐Jun* and activate MAPK signalling pathway via suppressing miR‐148a‐3p. We discovered gene‐*c‐Jun* was high expressed in HCV infection cells according to heat map and miR‐148a‐3p was lowly expressed. The forced overexpression of miR‐148a‐3p in vitro could suppress cell proliferation, block cell cycle in G1 stage and induce cell apoptosis.

During the past three decades, gene‐*c‐Jun* has been an interesting topic of hot investigation in the AP‐1 family. Early in 1990s, *c‐Jun*/*AP‐1* was found involved in various cellular responses.^17^, ^18^ In 1999, Leppa and Bohmann had summarized that the effects of *c‐Jun* were decided by the cell type. They also pointed out the role of the mitogen‐activated protein kinase (MAPK) signalling pathways played in the activity of AP‐1 factors.^19^ Now, *c‐Jun* has been proved to have a regulation on proliferation, apoptosis, tumorigenesis and many other cell activities in various cancers.^20^, ^21^ Previous study figured that *c‐Jun* mostly was a positive regulator in cell proliferation.^22^ Our findings were same as above. *C‐Jun* was up‐regulated in HCV‐infected hepatoma cells and had a regulation on cell activities—enhancement in cell proliferation and restraint in G0/G1 phase and apoptosis. It was well known that many proteins related to HCV took effect in cell processes through signaling pathways. Among them, MAPK was considered as a virion associated kinase and took effect in the human immunodeficiency virus infectivity at an early stage.It was well known that many proteins related to HCV took effect in cell processes through signalling pathways. Among them, MAPK was considered as a virion‐associated kinase and took effect in the human immunodeficiency virus infectivity at an early stage

Further, we investigated the potential targeted microRNA of *c‐Jun*. According to the pre‐existing studies, micro‐RNAs (miRNAs), small noncoding RNAs, play a predominant role in the control of gene expression.^13^, ^14^ In our study, we focused on hsa‐miR‐148a‐3p, which was lowly expressed in HCV infection host and had a targeted relationship with *c‐Jun*. Numerous studies have been performed to uncover miR‐148a‐3p was involved in several cancers. Lifers revealed miR‐148a, down‐regulated in human pancreatic ductal adenocarcinomas, regulated cell survival via CDC25B, a conserved dual specificity phosphatase which was significant in appropriate cell cycle.^23^ Similar results were also carried out in gastric cancer and HCC.^14^Yuan et al suggested that miR‐148a had a central role in hepatitis B virus (HBV) infected HCC via HBx, a trans‐activating protein related to the HCC development,^24^ and miR‐148a was found up‐regulated in glioblastoma and chordomas.^14^ In our study, we figured *c‐Jun* as the target protein of miR‐148a, and it was the first time to combine miR‐148a and *c‐Jun*. Moreover, overexpression of miR‐148a could restrain the expression of *c‐Jun*.

Finally, we investigated the regulation of miR‐148a on cell processes. Data showed that miR‐148a could inhibit proliferation, block G0/G1 phase in cell cycle and enhance cell apoptosis. The regulation was same with some other studies. Results of Bhattacharya et  al made it clear that reduction in miR‐148a led to the restraint of osteosarcoma cell death.^16^ Wang et  al revealed that miR‐148a could suppress proliferation in bladder cancer.^15^

Our study displayed a new kind of mechanism of *c‐Jun* overexpression and MAPK signalling pathway activation in HCV infection host—via regulating the expression miR‐148a. MiR‐148a and *c‐Jun* have been hot topics because of their various function. However, the mechanisms are complicated and not completely clear. In another word, more intensive researches are needed in this field. In a word, miR‐148a‐3p could suppress MAPK signalling pathway by inhibiting the expression of c‐Jun, and alleviated the progress of HCC infected with HCV.

## CONFLICT OF INTERESTS

The authors confirm that there are no conflict of interests.

## AUTHOR CONTRIBUTIONS

YBD, JCW, MJH and WJW substantially contributed to the conception and design of the work. MJH, GDX and WJW analysed and interpreted the data. YBD, JCW, MJH and HJQ drafted the manuscript. YBD and HJQ revised the work critically for important intellectual content. YBD and HJQ involved in the collection of grants. All authors made the final approval of the work.
