# Supplementary figures and images for: Inhibition of miR‐148a‐3p resists hepatocellular carcinoma progress of hepatitis C virus infection through suppressing c‐Jun and MAPK pathway
Source: J Cell Mol Med. 2018 Dec 18;23(2):1415–26. doi: 10.1111/jcmm.14045 (PMC6349179; doi:10.1111/jcmm.14045)

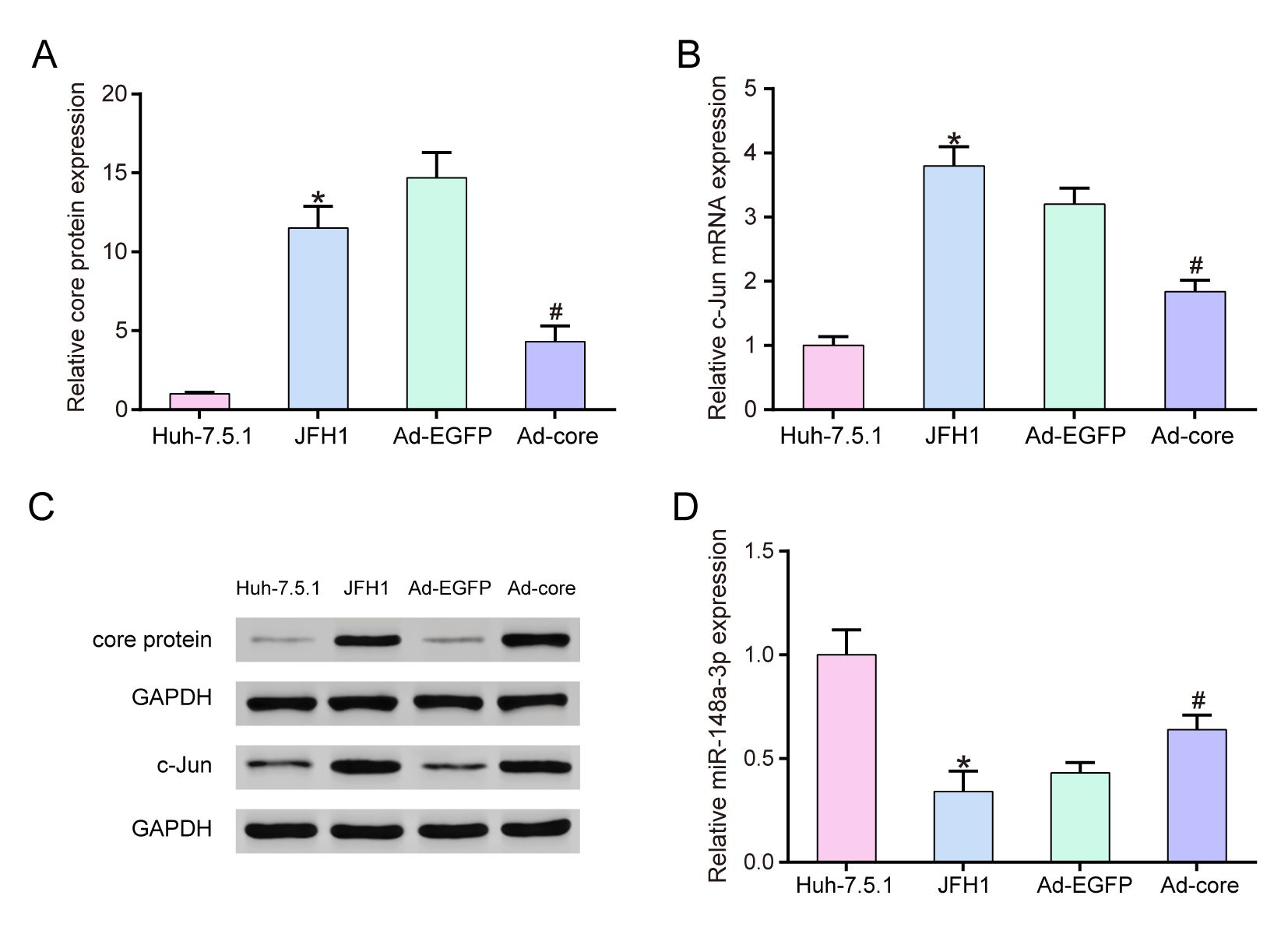

Supplement: Supplementary file 1 [file JCMM-23-1415-s001.tif]
