# Supplementary material for: Inhibition of miR‐148a‐3p resists hepatocellular carcinoma progress of hepatitis C virus infection through suppressing c‐Jun and MAPK pathway
Source: J Cell Mol Med. 2018 Dec 18;23(2):1415–26. doi: 10.1111/jcmm.14045 (PMC6349179; doi:10.1111/jcmm.14045)
Supplement: Supplementary file 2 [file JCMM-23-1415-s002.docx]

**Supplement table 1. Characteristics of patients with HCV infection and normal individuals**

| Parameter | Normal individuals  (n=15) | Hepatitis with HCV  (n=15) | HCC with HCV  (n=15) |
| --- | --- | --- | --- |
| Age (years) |  |  |  |
| <40 | 6 | 7 | 5 |
| ≥40 | 9 | 8 | 10 |
| Gender |  |  |  |
| Male | 8 | 9 | 11 |
| Female | 7 | 6 | 3 |
| Tumor size (cm) |  |  |  |
| <3 | N/A | N/A | 6 |
| ≥3 | N/A | N/A | 9 |
| AST (U/L) | 31±14 | 52±15 | 96±32 |
| ALT (U/L) | 28±12 | 45±18 | 73±26 |

N/A: not applicable
